# Bacterial Composition and Metabolomics of Dental Plaque From Adolescents

**DOI:** 10.3389/fcimb.2021.716493

**Published:** 2021-07-30

**Authors:** Kristian Havsed, Malin Stensson, Henrik Jansson, Miguel Carda-Diéguez, Anders Pedersen, Jessica Neilands, Gunnel Svensäter, Alex Mira

**Affiliations:** ^1^Department of Pediatric Dentistry, Institute for Postgraduate Dental Education, Jönköping, Sweden; ^2^Centre for Oral Health, School of Health and Welfare, Jönköping University, Jönköping, Sweden; ^3^Section for Oral Biology and Pathology, Faculty of Odontology, Malmö University, Malmö, Sweden; ^4^Folktandvården Skåne, The Swedish Dental Service of Skåne, Lund, Sweden; ^5^Department of Periodontology, Faculty of Odontology, Malmö University, Malmö, Sweden; ^6^Department of Health & Genomics, Foundation for the Promotion of Health and Biomedical Research (FISABIO) Foundation, Valencia, Spain; ^7^Swedish NMR Centre, The University of Gothenburg, Gothenburg, Sweden; ^8^Biofilms - Research Center for Biointerfaces, Malmö University, Malmö, Sweden

**Keywords:** *Streptococcus mutans* (*S. mutans*), NMR, metabolomics (OMICS), *Rothia*, acid tolerance, caries risk (assessment), dental caries, microbiome

## Abstract

Supragingival dental plaque samples were collected from 40 Swedish adolescents, including 20 with caries lesions (CAR) and 20 caries-free (CF). Fresh plaque samples were subjected to an *ex vivo* acid tolerance (AT) test where the proportion of bacteria resistant to an acid shock was evaluated through confocal microscopy and live/dead staining, and the metabolites produced were quantified by ^1^H Nuclear Magnetic Resonance (^1^H NMR). In addition, DNA was extracted and the 16S rRNA gene was sequenced by Illumina sequencing, in order to characterize bacterial composition in the same samples. There were no significant differences in AT scores between CAR and CF individuals. However, 7 out of the 10 individuals with highest AT scores belonged to the CAR group. Regarding bacterial composition, *Abiotrophia*, *Prevotella* and *Veillonella* were found at significantly higher levels in CAR individuals (p=0.0085, 0.026 and 0.04 respectively) and *Rothia* and *Corynebacterium* at significantly higher levels in CF individuals (p=0.026 and 0.003). The caries pathogen *Streptococcus mutans* was found at low frequencies and was absent in 60% of CAR individuals. Random-forest predictive models indicate that at least 4 bacterial species or 9 genera are needed to distinguish CAR from CF adolescents. The metabolomic profile obtained by NMR showed a significant clustering of organic acids with specific bacteria in CAR and/or high AT individuals, being *Scardovia wiggsiae* the species with strongest associations. A significant clustering of ethanol and isopropanol with health-associated bacteria such as *Rothia* or *Corynebacterium* was also found. Accordingly, several relationships involving these compounds like the Ethanol : Lactate or Succinate : Lactate ratios were significantly associated to acid tolerance and could be of predictive value for caries risk. We therefore propose that future caries risk studies would benefit from considering not only the use of multiple organisms as potential microbial biomarkers, but also their functional adaptation and metabolic output.

## Introduction

Microorganisms in the oral biofilm can metabolize dietary carbohydrates to produce organic acids, which will decrease pH and initiate demineralization of dental hard tissues (Marsh, 1994). *Streptococcus mutans* has for decades been considered the key pathogen for dental caries (Loesche et al., 1975), due its well-known acidogenic/aciduric properties as well as its ability to attach to enamel. However, during the last decades there has been a switch from explanations focused on specific-bacteria to an ecology-centred hypothesis of the disease (Marsh, 1994). In addition, other bacteria, ranging from *Bifidobacterium* to *Scardovia wiggsiae* (Kressirer et al., 2017) have also been found to be involved in tooth decay, and high-throughput sequencing data underline that caries is a polymicrobial disease (Simón-Soro and Mira, 2015). This hampers the strategy of using individual species as predictors of caries risk (Mira, 2018), which has been the classical approach with cultures of mutans streptococci or lactobacilli, and a global analysis of salivary microbiota has been proposed as an alternative (Teng et al., 2015). Other authors have advised that microbial analysis of oral samples should assess not only the taxonomic composition but also their functional features (Takahashi, 2015; Nascimento et al., 2017).

To identify key biomarkers for assessing bacterial composition and metabolic functions related to caries development, metatranscriptomic, proteomic, or metabolomic approaches can be used (Nyvad et al., 2013). For instance, metabolomic analysis of plaque samples after a sugar rinse has been performed by mass spectrometry, identifying key metabolites produced as a consequence of sugar fermentation (Takahashi et al., 2010). Recently, ^1^H Nuclear Magnetic Resonance spectroscopy (NMR) has been applied to identify a wide range of metabolites in oral samples, especially saliva (Gardner et al., 2018; Pereira et al., 2019). NMR is inherently quantitative and allows the detection and quantification of organic acids, alcohols and other metabolites in a small sample volume (Bertram et al., 2009). However, analyses of saliva samples will show metabolites produced by all oral communities including the tongue, palate, or epithelial mucosa among others, and metabolomic analysis of dental plaque samples would therefore be more informative in relation to caries development. Thus, the recent development of standardized *ex vivo* tests of acidogenicity and acid tolerance of dental plaque (Senneby et al., 2017) could provide a unique opportunity to study the metabolomic profile of caries-associated microbial communities. Adolescents may have a higher sensitivity for caries compared to other age-groups, as this period in life involves physical and psychological maturation together with a number of new teeth (Silk and Kwok, 2017). To our knowledge, research comparing bacterial composition and metabolomic data in adolescents is lacking, compared to the larger availability of data in adults and in childhood caries.

Thus, the aim of the present study is to compare bacterial composition, acid tolerance and metabolic output of oral bacteria in dental plaque samples, collected from adolescents with and without caries experience. To address this mixed taxonomic and functional analysis, we used a combination of NMR, high-throughput DNA Illumina sequencing and fluorescence microscopy on the same plaque samples, with the aim of understanding the interactions between bacteria and their metabolic products, as well as relating their composition, acidogenicity and acid tolerance to the risk of developing dental caries.

## Materials and Methods

### Study Design and Patient Selection

Forty subjects, aged 14-18 years, were selected (50% males). The participants received routine treatment at two Public Dental Services clinics, which were selected in areas with highest caries prevalence in adolescents, according to the statistics from Public Dental Service, Diver, in the county of Jönköping, Sweden. Ethical approval was obtained from the Regional Ethics Committee for Human Research at Linköping University, Sweden 2017/599-31 and from Swedish Ethical Review Authority, with reference 2019-05656. An intraoral examination included radiographic examination, registration of caries lesions and dental plaque index. All participants had permanent molars in occlusions and exclusion criteria included antibiotic treatment within three months, routine use of oral antiseptics, daily smoking, or regular use of snuff, and systemic or autoimmune diseases.

Study participants included a group of 20 individuals with caries lesions (CAR) and 20 who were caries-free (CF). Inclusion criteria in the caries-free group were no manifest or initial caries lesions and no past history of caries (DMFT index=0). Participants in the caries group had ≥3 surfaces with initial or manifest proximal and/or buccal caries lesions and a past history of caries (fillings). All participants were examined by the same specialist in pediatric dentistry. Initial and manifest caries were registered (clinical and radiographic) according to the criteria by Koch, 1967 and Alm et al., 2007. For intra-individual calibration, all bitewings were reviewed after an interval of three months. The weighted kappa value was 0.61 (CI 0.54-0.68). The percentage agreement was 89.7%.

### Sample Collection

Sampling was performed between October 2018 to February 2019. All participants were instructed to avoid tooth brushing from the night before sampling. Plaque was obtained by using a sterile Quickstick (Dab Dental AB) at interproximal sites in molar and premolar areas (3 sites per quadrant, 12 sites per individual). Sampling was avoided if any tooth was missing or if it didn´t exist any contact point between neighboring teeth. Interproximal plaque samples were pooled to give one final sample for each participant. Samples were added to a separate sterile microfuge tube, sealed, and sent on cold conditions to the laboratory within 24h. Upon arrival, biofilms were suspended in 500 µl sterile H_2_O and vortexed at 2500 rpm for 30 s (Mini analog vortex mixer, VWR, USA) to disperse the biofilms into individual cells before further analysis (Senneby et al., 2017).

### Plaque Acid Tolerance Test

Plaque acid tolerance was evaluated using a previously validated method (Neilands et al., 2012; Senneby et al., 2017). Briefly, 25 µl plaque sample was mixed with 75 µl TYE medium (1.7% tryptone, 0.3% yeast) containing 20 mM glucose and 40 mM phosphate/citrate buffer adjusted to pH 3.5 and incubated aerobically at 37°C for 2 hours. Following incubation, the cells were stained with LIVE/DEAD^®^ BacLight™ Fluorescent Stain (Molecular Probes) and transferred into an Ibidi mini flow cells (Ibidi GmbH). Flow-cells were then viewed with confocal laser scanning microscopy (CLSM) using a Nikon Eclipse TE2000 microscope (Nikon Corp.) with an Ar laser (488 nm laser excitation). Images were acquired with a Photometrics Prime 95B camera using Nikon NIS-Elements software. Ten randomly selected images from each sample were saved for further analysis. All confocal images were examined by an experienced oral microbiologist and given a score 1-5 as described by Senneby et al. (2017). This method has been shown to have high intra-rater agreement and the scoring corresponding well to the percentage obtained by manually counting the cells (Senneby et al., 2017).

### Generation of Metabolic End Products

Two hundred µl of the suspended plaque sample was diluted in 37°C 1,2% NaCl solution to a final concentration of 0,9% NaCl. One molar sterile glucose solution was added giving a final concentration of 100 mM glucose. This was followed by incubation at 37°C for 60 min. After incubation, the sample was cooled on ice for minimum 5 min, then centrifuged at 14000 rpm for 10 min. Supernatants were stored at -80°C until further analysis. Metabolomic analysis of plaque supernatants were obtained by NMR.

### Metabolomic Analysis of Dental Plaque: NMR Data Acquisition and Processing

Plaque supernatants were thawed at room temperature for 20 min and spun down at 2000xg, 4°C for 5 min in a S-4-72 swing-out rotor of a 5804R centrifuge (Eppendorf). 180 µl supernatant was added to 2 ml cryo vials (Sarstedt) containing 20 µl buffer (1.5M potassium phosphate, pD 6.95, in 100% D2O, 0.5% w/v sodium azide and 0.1% w/v 3-(trimethylsilyl)propionic-2,2,3,3-d4 acid (TSP-d4)). Similarly, one blank sample (0.1 M glucose, 0.9% w/v sodium chloride) was made the same way and also a buffer-only sample, exchanging sample volume with LC-MS-grade water. The cryovials were put into a rack and shaken at 800 rpm for 2 min at 12°C in a Thermomixer Comfort (Eppendorf). 180 µl of each sample was transferred to a 3 mm SampleJet NMR tube rack using a Bruker SamplePro Tube L liquid handling robot, keeping samples and NMR tube rack at 2°C throughout the preparation. The SampleJet rack was put in a cooled SampleJet sample changer on an 800 MHz Oxford magnet equipped with a Avance III HD console and a 3 mm TCI cryoprobe (Bruker BioSpin). 1D ^1^H NMR data was acquired using the ‘zgespe’ pulse sequence, encompassing water suppression through excitation sculpting with a perfect echo element. 128 scans were acquired into 65536 data points with a spectral width of 20 ppm, acquisition time of 2.04 s, inter-scan relaxation delay of 3 s and a fixed receiver gain of 18. The low receiver gain setting was necessary to avoid analog/digital converter overflow stemming from the intense glucose signals. TopSpin3.5pl7 (Bruker BioSpin) was used for acquisition and processing of data. Data was Fourier-transformed, including zero-filling twice, 0.3 Hz line-broadening, phasing, baseline correction and referencing to TSP-d4. Processed data was imported into MatLab 2017b (MathWorks Inc.), aligned with icoshift (Savorani et al., 2010) and integrated manually peak-by-peak down to a linear baseline using an in-house developed routine. Peak annotation was accomplished with ChenomX 8.4 (Chenomx Inc.) and the Human Metabolome Database (HMDB) (Wishart et al., 2018). All NMR data on an individual basis are provided as a **Supplementary Dataset**.

### NMR Data Correlation Analyses

In order to compare the abundance of detected metabolites, data (arbitrary units, au) were normalized by ng of plaque’s DNA. We used Sparse Partial Least Squares (sPLS) (canonical mode) as a multivariate methodology to perform simultaneous variable selection in the two datasets (the species abundance and the NMR output) based on the importance of the association computed (Lê Cao et al., 2009). These associations were plotted in a heatmap and a network using the mixOmics R package (Rohart et al., 2017).

### Sequencing of the 16S rRNA Gene

DNA from plaque samples was extracted using the MagNa Pure LC DNA Isolation kit II in a MagNa Pure Robot (Roche), following the protocol recommended by the manufacturer with some modifications (Dzidic et al., 2018). DNA concentration was estimated with the Quant-iT™ PicoGreen^®^ dsDNA Assay Kit and a Qubit™ 3 Fluorometer (ThermoScientific). The V3-V4 hypervariable region of the 16S rRNA gene was amplified using the universal primers V3-V4 Forward (CCTACGGGNGGCWGCAG) and V3-V4 Reverse (GACTACHVGGGTATCTAATCC) optimized for Illumina sequencing. Library was constructed using the Metagenomic Sequencing Library Preparation Illumina protocol (Part #15044223 Rev. A) and sequenced at the FISABIO Institute (Valencia, Spain) using 2x300 bp paired-end sequencing with an Illumina MiSeq instrument. Sequencing data have been publicly deposited in the SRA database (Bioproject PRJNA681486, Accession number SRR13194555- SRR1319594).

### Bioinformatic Analysis of Sequencing Data

The software Dada2 v1.16 was used to filter, end-trim, denoise and merge paired reads (Callahan et al., 2016) using default parameters. Adapters and primers were first filtered out from the sequence reads and then end-trimmed in 10 bp windows with quality values <35 and absence of Ns. Singletons reads were removed except for calculating richness and diversity indexes. The remaining reads were merged, clustered and cleaned for host and chimeric reads and finally assigned a taxon at the genus and species level (with Amplified Sequence Variants, or ASVs) using the SILVA non-redundant database v138.1 (Quast et al., 2013).

We used overall R programming language for statistical computing (R Development Core Team, 2016) to perform downstream analyses. Genera with an abundance of <0.01% were removed from all samples. For multivariant analysis, an Adonis test (Permutational Multivariate Analysis of Variance Using Distance Matrices), provided by the Vegan library of R (Oksanen et al., 2015), was used to compare groups. Rarefaction curves, richness and diversity indexes were performed with 20,000 sequences per sample. To visualize groups and their differences in a two-dimensional map, we computed constrained correspondence analysis (CCA) with the Vegan library (Oksanen et al., 2015). For univariate analyses, non-parametric Wilcoxon tests (wilcox.test function of stats library of R) (R Development Core Team, 2016) were performed to test the differences in genera and species-level taxa. Adjusted p-values, obtained by the FDR method, were used.

We followed Random Forest modelling (Liaw and Wiener, 2002) to address complex interactions between bacterial biomarkers, in order to determine the number of bacteria necessary to efficiently discriminate between CAR and CF groups. We used the bootstrapping algorithm implemented in Boruta R library (Kursa and Rudnicki, 2010) to select biomarkers. The model accuracy was evaluated by the area under curve (AUC) values, when using 30 or less bacteria with the top biomarker scores, ending by the diagnostic value with the top 3 biomarkers (a random forest model needs at least 3 variables). This analysis was performed for biomarkers at the genus and species levels.

## Results

The mean age of participants was 16.3 and 15.6 years in CAR and CF groups. Three different analyses were performed for each dental plaque sample: 16S rRNA gene sequencing, acid tolerance test and metabolomics. All participants in the caries group exhibited proximal initial caries lesions (range 1-16 surfaces) and ten participants had proximal manifest cavities (0-12 surfaces). Mean and median carious tooth surfaces including initial and manifest caries lesions and dental restorations (D_i+m_F) were 17.25 and 15.00 (see **Supplementary Table 1** for details).

### Oral Microbiota Associated to Dental Plaque

**Figure 1:**
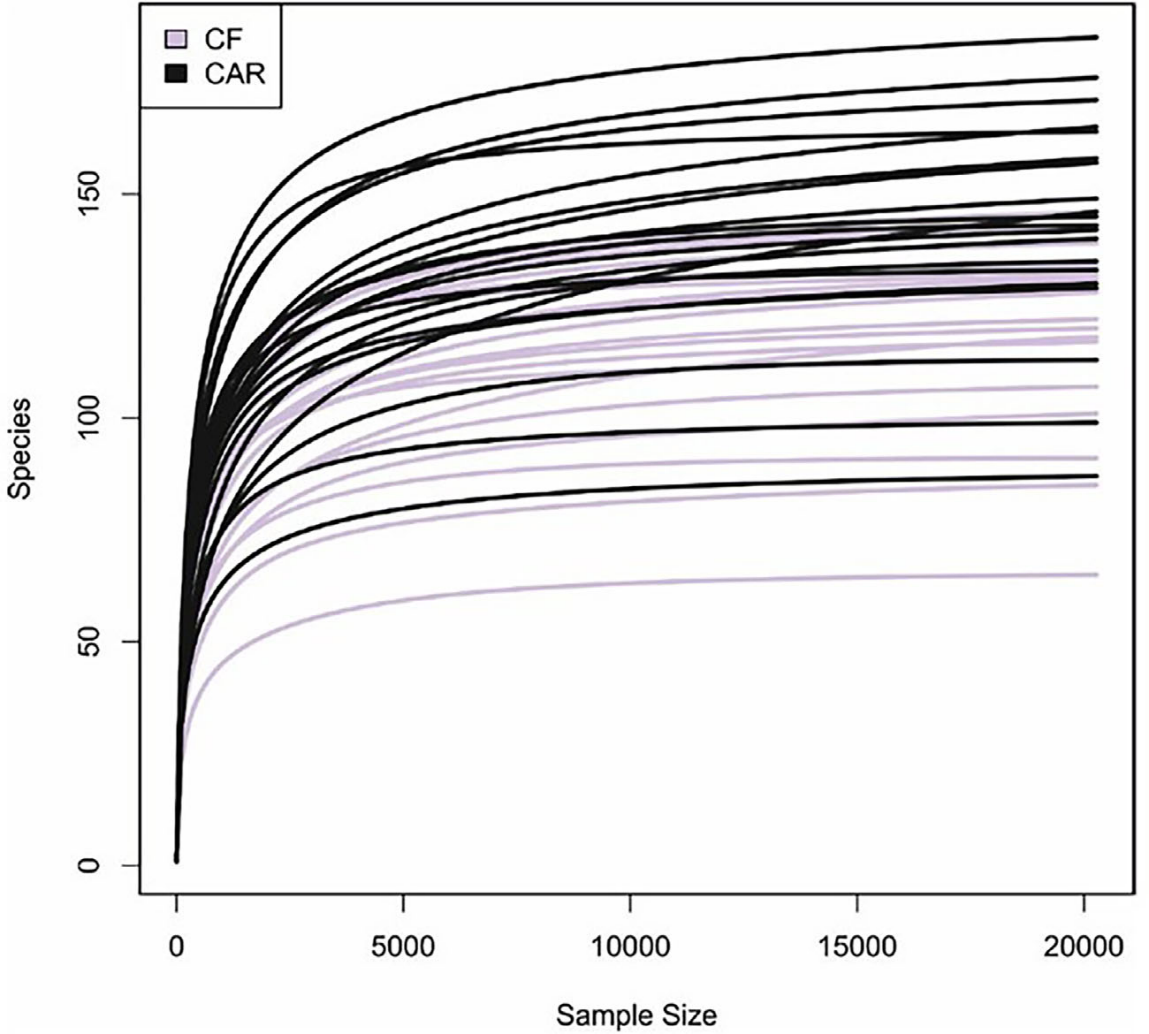
Rarefaction curves of caries (CAR) and caries-free (CF) individuals. The estimated number of bacterial species detected are plotted relative to the number of sequence reads obtained by Illumina sequencing of the 16S rRNA gene. Bacterial richness is stabilized at 20.000 reads (minimum number of reads for all samples).

**Figure 2:**
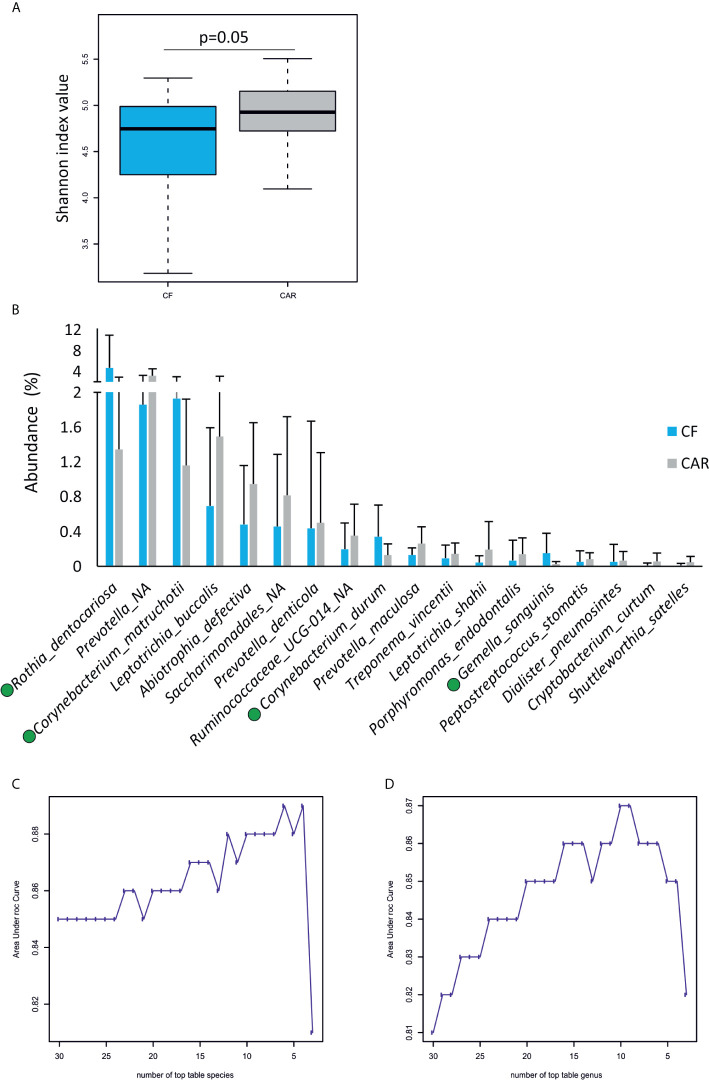
Microbiology of dental plaque samples from caries-experienced (CAR) and caries-free (CF) adolescents, as obtained by sequencing of the 16S rRNA gene. **(A)** Boxplots show diversity (Shannon) and richness (Chao1) indexes in CAR and CF individuals. The p-values (Wilcoxon test) are indicated. **(B)** Mean abundance of bacterial species found at significantly different proportions in either CF or CAR groups (p-values <0.05, Wilcox test). Bacteria over-represented in CF individuals are highlighted by a green dot. Lower plots represent the accuracy (Area under Curve) of random forest models to discriminate between CAR and CF individuals according to the number of bacterial species **(C)** and genera **(D)**.

**Figure 3:**
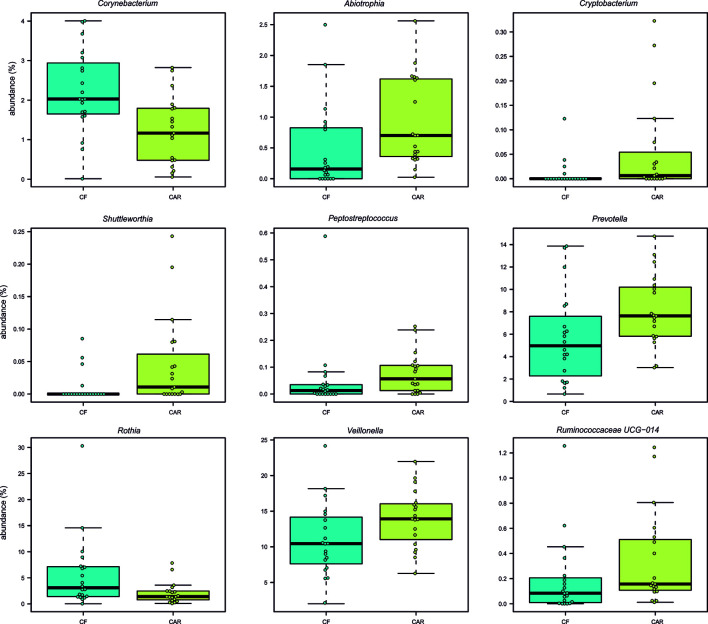
Bacterial genera with statistically significant differences in abundance between caries-free (CF) and caries-experienced (CAR) adolescents. Individual samples are represented as circles. All p-values (Wilcox test) are lower than 0.05.

After trimming, quality filtering and annotation of the reads the mean number of sequences per sample was 70,266 ± 6,539. According to the rarefaction curves, all samples showed saturation of diversity after 10.000 reads, suggesting that the sequencing effort was sufficient to cover diversity at species level (**Figure 1**). Bacterial composition was similar to what had previously been described in other populations (**Supplementary Figure 1**). We detected higher but not significant mean values for richness (as quantified by the Chao1 index) and statistically significant higher diversity values (Shannon index, p=0.05) in the CAR group (**Figure 2A**). At genus level, there was a significantly higher proportion of *Rothia* and *Corynebacterium* and lower abundances of *Abiotrophia*, *Cryptobacterium*, *Shuttleworthia*, *Peptostreptococcus*, *Prevotella*, *Veillonella* and *Ruminoccocaceae UCG-014* in CF individuals (**Figure 3**). At species level, *Rothia dentocariosa, Corynebacterium matruchotii, Corynebacterium durum* and *Gemella sanguinis* were significantly more abundant in CF individuals (**Figure 2B**). Meanwhile, 14 species were associated to CAR individuals, including a not assigned *Prevotella*, *Leptotrichia buccalis, Abiotrophia defectiva*, unassigned *Saccharimonadales* or *Prevotella denticola.* In addition, we used a random forest analysis to build a model that predicts how many features (genera or species) are needed to differentiate a CAR from a CF individual (**Figures 2C, D** **)**. As a result, a minimum of 4 species and 9 genera are necessary to differentiate these two groups with an accuracy of 0.87 or higher. Therefore, not a single species could be detected as a robust biomarker discriminating the caries status of participants, due to overlap between the two groups, even for *Abiotrophia* which presents the most significant difference. The best studied caries-associated bacterium to date, *Streptococcus mutans*, was found at extremely low prevalence (8 out of 20 individuals in the CAR group and 2 out of 20 individuals in the CF group). Its mean frequency in the CAR group was 0.16% (and 0.003% in the CF group), showing that although it could be a potential biomarker of disease, it is still absent in more than 50% of the studied CAR adolescents.

### Acid Tolerance Test

**Figure 4:**
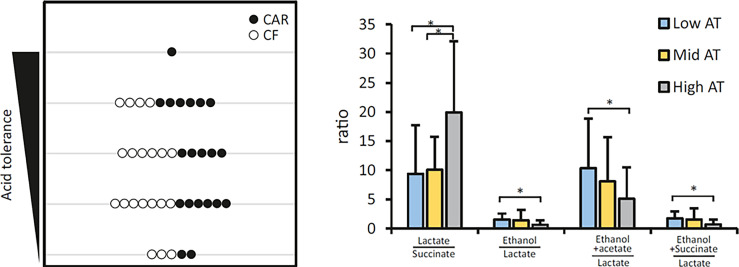
Acid Tolerance (AT) of dental plaque from adolescents. The left panel represents the distribution of caries-experienced (CAR) and caries-free (CF) individuals in different acid tolerance groups. Individuals are assigned to an acid tolerance group from 1 to 5, being 1 the lowest and 5 the highest acid tolerance detected in dental plaque samples exposed to high sugar conditions. Bar graphs on the right panel represent the means of several metabolite ratios for Low (AT levels 1-2), Intermediate (AT level 3) and High (AT levels 4-5) acid tolerance groups. Statistically significant differences are indicated by an asterisk. Metabolite profiles in plaque supernatants exposed to high sugar conditions were obtained by NMR analysis.

As explained before (Senneby et al., 2017), an Acid Tolerance (AT) test allows us to separate individuals into 5 groups depending on their Acid Tolerance level (1 to 5 AT value) being 1 those with a small proportion of acid tolerant bacteria and 5 those with a large proportion. Although 7 out of 10 individuals with high AT scores were CAR, no significant differences in AT were detected between CAR and CF individuals (**Figure 4**, left panel). However, we did detect differences in the proportions of some specific bacteria. To clarify the differences, groups with the lowest (AT values 1 and 2) and those with the highest tolerance (AT values 4 and 5) were pooled. When these two clusters were compared, several species showed differences in abundance (**Supplementary Figure 2**). *Fusobacterium periodonticum, Prevotella melaninogenica, Campylobacter concisus*, and unassigned species of *Veillonella* and *Alloprevotella* were significantly more abundant in the individuals with higher acid tolerance. *Streptococcus salivarius, Fusobacterium nucleatum* and *Campylobacter gracilis* were more prevalent in the plaque samples that had lower AT. Interestingly, different *Fusobacterium* and *Campylobacter* species were associated with different clusters, underlining the importance of species-level resolution analysis.

### Metabolomic Profile

A total of 67 peaks or peak clusters were aligned and integrated out of which 39 could be unequivocally annotated. The rest were putatively annotated or not assigned and numbered in ascending order. Using normalized data, the most abundant metabolites detected (besides glucose) in the samples were acetate (14.2 ± 17 Kau/ng), metabolite 16 -putatively assigned to lactate-proline mix- (6.9 ± 3.8 Kau/ng), lactate (2.6 ± 2.2 Kau/ng), metabolite 44 (1.8 ± 2.3 Kau/ng) and ethanol (1.8 ± 1.4 Kau/ng). When the concentration of the metabolites was compared between CAR and CF groups, 3-hydroxyisovalerate appeared to be over-represented in the CF samples, but this difference disappeared when using corrected p-values (**Table 1**). In addition, metabolites 28 (putatively assigned to glutamate-proline) and 25 showed a statistical trend (0.05<p<0.1) but again only when using uncorrected p-values. However, when the metabolites were compared between Low and High AT clusters the number of metabolites differentially represented increased up to 10 and an additional 4 showed a statistical trend. Interestingly, all these 14 metabolites were more abundant in the Low AT group.

**Table 1:** Metabolites significantly associated with caries status or with the acidogenicity of plaque.

Caries Status	p-value	adj. p-value	CF (au/ng)	CAR (au/ng)	log_2_FC
3-mutayisovalerate	0.04	0.83	817.12	477.34	0.76
25	0.07	0.83	526.98	537.13	-0.03
28	0.096	0.83	414.17	194.37	1.05
**Acid Tolerance**	**p-value**	**adj. p-value**	**Low (au/ng)**	**High (au/ng)**	**log_2_FC**
ethanol	0.0002	0.01	2213.27	803.89	1.45
succinate	0.0002	0.01	295.71	114.20	1.30
14	0.0014	0.03	1787.87	831.50	1.10
glucose	0.0025	0.04	624027.29	324501.47	0.94
isopropanol	0.01	0.06	793.65	103.43	2.83
15	0.01	0.08	1648.90	903.96	0.86
16	0.01	0.08	7738.91	4542.06	0.77
12	0.01	0.08	1580.78	920.77	0.77
3-hydroxyisovalerate	0.02	0.10	684.11	392.79	0.79
ethanol_isopropanol	0.03	0.16	273.71	83.84	1.60
45	0.06	0.33	529.27	336.90	0.64
21	0.08	0.35	194.30	79.88	1.19
propionate	0.09	0.39	256.69	159.64	0.65
40	0.099	0.39	1477.33	868.50	0.76

Au, arbitrary units; CF, Caries-Free individuals; CAR, Caries-experienced individuals; FC, Fold-Change.

Taking into consideration the metabolites that were significantly associated to Low AT group, we calculated several ratios that could help us to separate individuals with different AT capacity (**Figure 4**, right panel). Lactate/succinate, ethanol/lactate, ethanol+acetate/lactate and ethanol+succinate/lactate significantly separated High AT from Low AT samples. Moreover, lactate/succinate also had a significantly higher value in High AT than Mid AT.

### Microbiota-Metabolites Correlations

**Figure 5:**
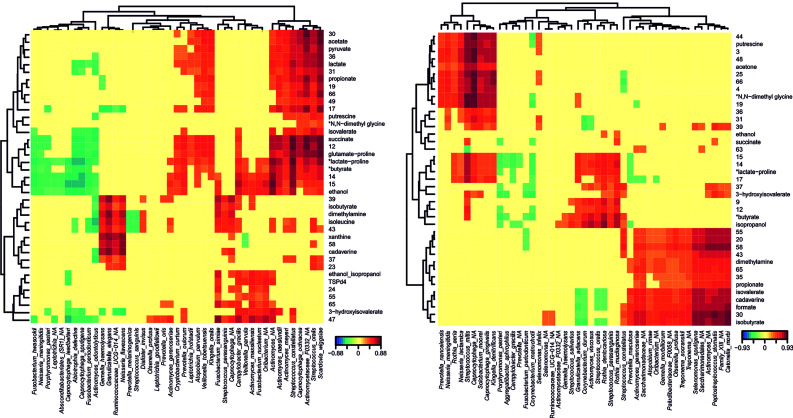
Metabolomic profiles of dental plaque samples from Caries-experienced (CAR) and Caries-Free (CF) individuals. NMR was used to quantify metabolites produced by dental plaque samples exposed to high sugar levels. The heatmaps show those metabolites with a significant correlation with bacterial species identified by Illumina sequencing of the 16S rRNA in CAR (left panel) and CF (right panel) individuals. The degree and sign of the correlations are represented by the color code shown below. Each bacterial species shows a metabolomic profile (columns), and those profiles were clustered according to their similarity (top and left dendrograms). Those metabolites for which the NMR peaks were not unique are marked with an asterisk next to the putative metabolite assigned.

To integrate available data, we studied the putative correlations between species abundances and metabolites concentrations for the different groups studied (CAR, CF, Low AT, Mid AT, and High AT), clustering those bacteria with a similar metabolomic profile in heatmap plots. Significant correlations for CAR and CF groups are shown in **Figure 3**. Clear clusters were formed by species that correlate significantly with a given set of metabolites (positively and negatively). For example, when correlations for CAR individuals are shown, several organic acids (acetate, succinate, and lactate) form a cluster with high positive correlations with *Streptococcus constellatus, Scardovia wiggsiae, Capnocytophaga ochracea, Veillonella tobetsuensis, Atopobium parvulum*, or *Actinomyces* sp. Interestingly, these organic acids do not seem to correlate significantly with any species in the CF group. However, in caries-free individuals, a cluster was formed with some oral microbes typically associated with oral health such as *S. oralis, S. parasanguinis, Corynebacterium durum, Rothia dentocariosa* or *R. mucilaginosa*, which correlate positively with isopropanol, 3-hydroxyisovalerate and succinate (among other unknown metabolites) (**Figure 5**).

**Figure 6:**
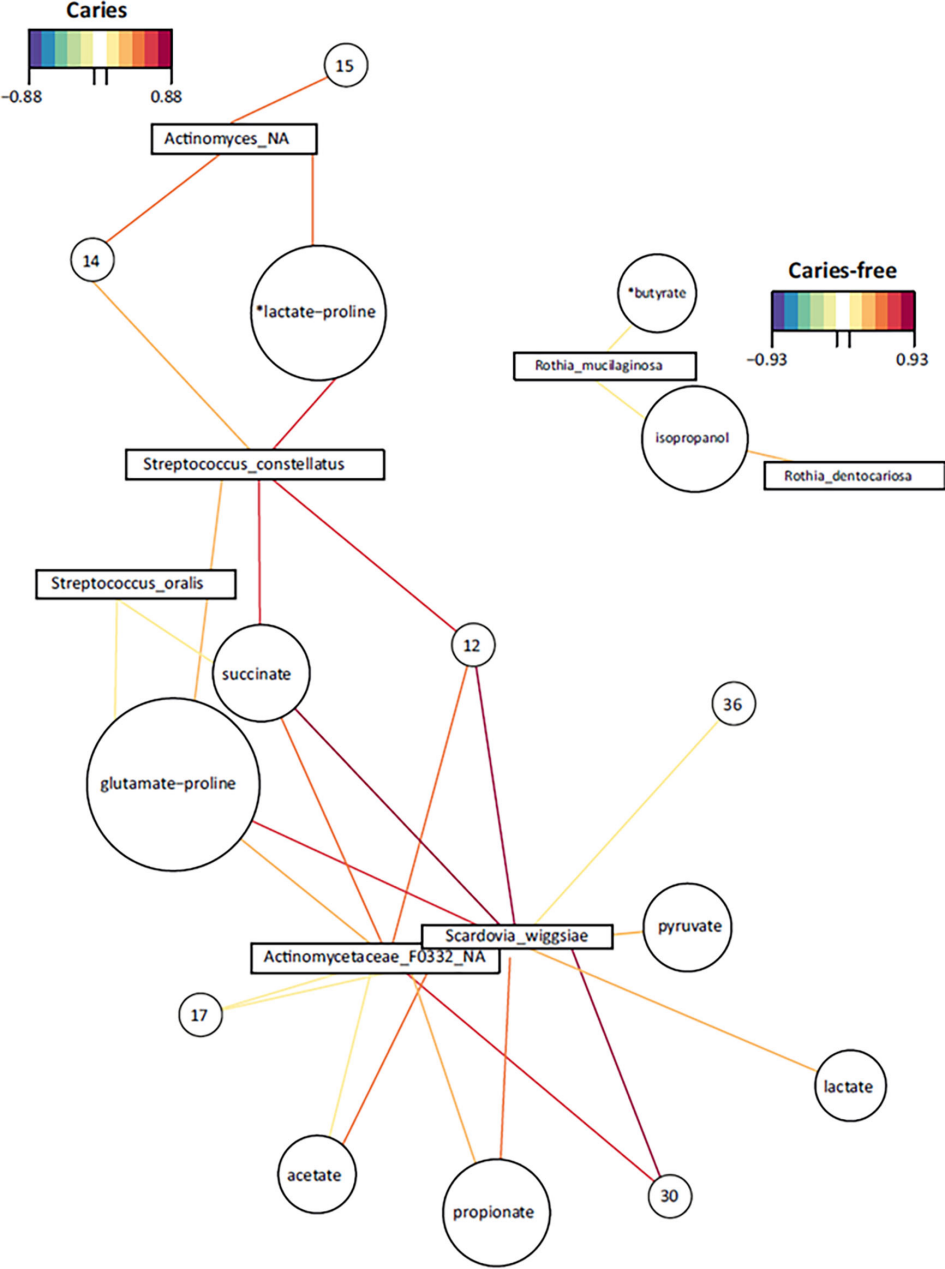
Bacteria-Metabolites networks in dental plaque samples. Representative networks with significant correlations from caries-experienced (CAR) and caries-free (CF) adolescents are shown. Positive and negative significant correlations between individual species (squares) and metabolites (circles) are represented with colored lines according to the palettes. The size of the circles is arbitrary. Metabolites were identified by NMR analysis and those compounds with non-unique peaks are marked with an asterisk to indicate their putative assignment. Compounds marked with numbers could not be assigned to a known metabolite.

In addition, when a network using metabolites-bacterial correlations was built, several species highly correlated with organic acids among other molecules, and *Scardovia wiggsiae* appears to be central in this network (**Figure 6**). In CF individuals a cluster formed by *Rothia* species showed a correlation with isopropanol and a metabolite putatively assigned to butyrate.

Regarding the correlation of bacterial species with metabolites in the AT groups, several clusters were also apparent (**Supplementary Figure 3**). In the Low AT group, three clusters were formed. In one of them, several health-associated, nitrate-reducing organisms are observed such as *Kingella denitrificans*, *Neisseria lactamica* or *Rothia aeria* which all correlate with acetone and some unknown metabolites. A second cluster correlated with succinate, ethanol, ethanol-isopropanol, dimethylamine, isobutyrate and isovalerate. In the microbiota associated with high AT, lactate, acetate, pyruvate, xanthine, formate, and dimethylamine formed a cluster with positive correlations with some putative oral pathogens such as *G. morbillorum*, *Parvimonas micra*, *Leptotrichia shahii*, *Porphyromonas catoniae*, *Selenomonas noxia* or *Treponema socranskii*.

## Discussion

The results obtained in the current manuscript regarding bacterial composition are consistent with the ecological hypothesis of dental caries (Marsh, 1994) and with a polymicrobial etiology of the disease (Simón-Soro and Mira, 2015). The prevalence of *S. mutans*, which has long been considered the main caries causing agent, was only 40% in our CAR samples, where it accounted for 0.16% of the total. Thus, although their mean proportion was higher than that observed in the CF group, it is not a universal marker for the disease (Mira, 2018). This is consistent with the work by Johansson and collaborators, who concluded that the role of mutans streptococci as primary caries pathogens is less pronounced in populations with prevention programs (Johansson et al., 2016), such as the one studied in our work. However, our data identify other bacteria with higher association to caries experience, including *Scardovia wiggsiae*, *Prevotella denticola* or *Abiotrophia defectiva*, all of which have previously been found at higher levels in caries-active children and to have strong acidogenicity (ElSalhy et al., 2016; Kressirer et al., 2017; Zhang et al., 2020). The higher levels of *Veillonella* in the caries group is probably related to its dependency on organic acids as carbon source, suggesting that this organism could be a good marker for high lactate levels (Belda-Ferre et al., 2012). Health-associated bacteria included *Corynebacterium*, which is now identified as a key species in biofilm architecture (Mark Welch et al., 2016), and *Rothia*. The latter are consistently associated with good oral health in multiple studies, and recent work has demonstrated its capacity to buffer extracellular pH by lactate use and ammonia production from dietary nitrate (Rosier et al., 2020).

Our metabolomic data show that *S. wiggsiae* has a central role in organic acid production and identify this species as a fundamental player in caries risk in adolescents, consistent with its already-established participation in childhood caries (Kressirer et al., 2017). However, it is not universally distributed in all caries-experienced individuals, again suggesting that caries etiology is not only polymicrobial but also highly variable among individuals (Mira, 2018). Previous studies targeting specific bacteria have demonstrated that models based on two organisms, such as *S. mutans* and *Lactobacillus* (Featherstone, 2000) or *S. mutans* and *Prevotella pallens* (Zhang et al., 2020) improve caries assessment compared to the use of a single species. Our open-ended modelling data, based on random forest classification, indicate that optimal models require at least four oral species or nine genera, and even with this high number of organisms, classification accuracy is below 90% (**Figure 1**). This suggests that apart from taxonomic classification, functional assessment of bacterial communities could be instrumental to predict caries risk.

A limitation of the current study is a modest sample size (n=20 individuals per group) and the use of incomplete 16S rRNA sequences (450 bp in length) derived from Illumina reads. The use of short length reads of the 16S rRNA gene has been shown to limit accurate taxonomic assignment at the species level (Claesson et al., 2010). Thus, although we have used an advanced bioinformatic method for assignment Amplicon Sequence Variants to bacterial species, some of these assignments may contain mistakes or just be impossible to be performed, especially in bacterial genera which present similar sequences among their corresponding species such as streptococci, where *S. mitis*, *S. infantis* or *S. dentisani* contain identical sequences in the amplified segment of the 16S rRNA gene analyzed by standard Illumina library protocols (Dzidic et al., 2018). The taxonomic data assigned at the genus level in the current manuscript are therefore more robust, but species-level analyses are also included given their congruence with the results from genus-level data and the high-resolution of the 16S rRNA gene sequence for many oral taxa. Future work should also consider performing whole-DNA metagenomic sequencing in order to provide a functional assessment of the gene content of caries and caries-free bacterial communities (Belda-Ferre et al., 2012) and studying the potential role of other members of the microbiome, such as fungi, which were not considered in the current manuscript. Another limitation is given by the high variability in caries experience among CAR individuals, and the absence of past caries experience in some participants from this group, which could make them overlap in physiological or microbiological features with caries-free participants. However, our work aimed at identifying biomarkers of caries in a common population, not only in those individuals at the extreme of the caries landscape, even if this implies that the microbial signals of tooth decay may be weak in those individuals with low caries burden.

Central to caries development is the capacity of supragingival biofilm bacteria to metabolize dietary carbohydrates to organic acids that when accumulated and dissociated disturb the delicate balance between enamel de- and remineralization. In the current study, plaque samples were exposed to an excess of glucose to simulate metabolism taking place at carbohydrate intake. NMR was found to be a powerful methodology for metabolite profiling, with over 60 spectral features from 39 unique metabolites assigned in our samples, despite the difficulty imposed by the high glucose content. A lower glucose concentration would not only enable increase of the receiver gain, allowing detection of less abundant metabolites, but also reduce the amount of overlap from the glucose signals, which cover the 3.3 to 3.9 ppm spectral region. Given that some health-associated peaks in the NMR profile could not be unequivocally identified (*e.g.* compounds 14, 15 or 17), future work should be aimed at identifying their nature and potential role in caries prevention. When considering caries-experiencing individuals, several species display highly significant correlations with organic acid production, including not only lactate but also propionate, acetate, or pyruvate (**Figure 5**), and a similar pattern was found in high-AT individuals (**Supplementary Figure 3**). The combined effect of some of those acids could be instrumental for caries development, as it has been demonstrated that acetic and lactic acid have additive demineralizing properties (Featherstone and Rodgers, 1981) and similar synergistic processes could be taking place for other combinations of organic acids. Interestingly, in both CF and low-AT groups, there are no significant correlations of any bacterial species with lactate. On the contrary, in these health-associated communities we identify significant associations with succinate, ethanol, isopropanol or acetone. Some of these associations are strong in nitrate-reducing species like *Neisseria*, *Rothia* or *Kingella*, supporting an important role of nitrate in oral health homeostasis (Rosier et al., 2018) but significant correlations are also found in other bacteria like *Streptococcus oralis*, *Capnocytophaga* spp. or *Corynebacterium durum*. The opposite trends shown for lactate and succinate, as well as for lactate and ethanol-isopropanol, are consistent with alternative fermentation pathways that could drive the selection of acid-tolerant over competing bacterial strains in caries and high-AT individuals (Marsh, 1994). A shift from lactate to ethanol production has also been observed in several oral bacteria under low glucose or low growth rate conditions (Mikx and van der Hoeven, 1975; Yamada and Carlsson, 1975). In agreement with these two metabolic paths driving high and low acidogenicity, lactate:succinate and lactate:ethanol ratios are significantly different between AT levels (**Figure 4**), in line with the altered metabolism towards lactate production by acid tolerant bacteria even when the environmental pH is lowered (Matsui and Cvitkovitch, 2010). Future studies should evaluate if these ratios could have a diagnostic and predictive value for caries disease.

## Data Availability Statement

The datasets presented in this study can be found in online repositories. The names of the repository/repositories and accession number(s) can be found below: https://www.ncbi.nlm.nih.gov/genbank/, SRR13194555- SRR1319594.

## Ethics Statement

The studies involving human participants were reviewed and approved by Regional Ethics Committee for Human Research at Linköping University, Sweden 2017/599-31 and from Swedish Ethical Review Authority, with reference 2019-05656. Written informed consent to participate in this study was provided by the participants’ legal guardian/next of kin.

## Author Contributions

AM: Performed sequencing work, contributed to conception, design, data acquisition and interpretation, and drafted and critically revised the manuscript. MS, HJ, GS: Contributed to conception, design, data acquisition and interpretation, and drafted and critically revised the manuscript. KH Contributed to conception and design, collected samples, performed dental examinations, and drafted and critically revised the manuscript. JN performed acid tolerance experiments, contributed to data analysis, and critically revised the manuscript. AP performed NMR experimental work, contributed to data analysis, and critically revised the manuscript. MC-D contributed to data analysis and designed all figures, performed statistical analysis, and critically revised the manuscript. All authors contributed to the article and approved the submitted version.

## Funding

This project was funded by grants 807631 and 931593**** funded by the Medical Research Council of Southeast Sweden; grant 931659 funded by**** Futurum-Academy for Health and Care, Jönköping County Council; grant 2016-01994 from The Swedish Research Council; and grant BIO2015-68711-R funded by the Spanish Ministry of Science and Innovation and FORESIGHT multidisciplinary programme at Malmö University. MC-D was funded by grant APOSTD 2018/081 from Generalitat Valenciana.

## Conflict of Interest

The authors declare that the research was conducted in the absence of any commercial or financial relationships that could be construed as a potential conflict of interest.

## Publisher’s Note

All claims expressed in this article are solely those of the authors and do not necessarily represent those of their affiliated organizations, or those of the publisher, the editors and the reviewers. Any product that may be evaluated in this article, or claim that may be made by its manufacturer, is not guaranteed or endorsed by the publisher.

## References

[B1] Alm A. Wendt L. K. Koch G. Birkhed D. (2007). Prevalence of Approximal Caries in Posterior Teeth in 15-Year-Old Swedish Teenagers in Relation to Their Caries Experience at 3 Years of Age. Caries Res. 41, 392–398. 10.1159/000104798 17713340

[B2] Belda-Ferre P. Alcaraz L. D. Cabrera-Rubio R. Romero H. Simón-Soro A. Pignatelli M.. (2012). The Oral Metagenome in Health and Disease. ISME J. 6, 46–56. 10.1038/ismej.2011.85 21716308 PMC3246241

[B3] Bertram H. C. Eggers N. Eller N. (2009). Potential of Human Saliva for Nuclear Magnetic Resonance-Based Metabolomics and for Health-Related Biomarker Identification. Anal. Chem. 81, 9188–9193. 10.1021/ac9020598 19780580

[B4] Callahan B. J. McMurdie P. J. Rosen M. J. Han A. W. Johnson A. J. A. Holmes S. P. (2016). DADA2: High-Resolution Sample Inference From Illumina Amplicon Data. Nat. Methods 13, 581–583. 10.1038/nmeth.3869 27214047 PMC4927377

[B5] Claesson M. J. Wang Q. O’Sullivan O. Greene-Diniz R. Cole J. R. Ross R. P.. (2010). Comparison of Two Next-Generation Sequencing Technologies for Resolving Highly Complex Microbiota Composition Using Tandem Variable 16S rRNA Gene Regions. Nucleic Acids Res. 38, e200–e200. 10.1093/nar/gkq873 20880993 PMC3001100

[B6] Dzidic M. Collado M. C. Abrahamsson T. Artacho A. Stensson M. Jenmalm M. C.. (2018). Oral Microbiome Development During Childhood: An Ecological Succession Influenced by Postnatal Factors and Associated With Tooth Decay. ISME J. 12, 2292–2306. 10.1038/s41396-018-0204-z 29899505 PMC6092374

[B7] ElSalhy M. Söderling E. Honkala E. Fontana M. Flannagan S. Kokaras A.. (2016). Salivary Microbiota and Caries Occurrence in Mutans Streptococci-Positive School Children. Eur. J. Paediatr. Dent. 17, 188–192. 27759406

[B8] Featherstone J. D. (2000). The Science and Practice of Caries Prevention. J. Am. Dent. Assoc. 131, 887–899. 10.14219/jada.archive.2000.0307 10916327

[B9] Featherstone J. D. Rodgers B. E. (1981). Effect of Acetic, Lactic and Other Organic Acids on the Formation of Artificial Carious Lesions. Caries Res. 15, 377–385. 10.1159/000260541 6942921

[B10] Gardner A. Parkes H. G. Carpenter G. H. So P.-W. (2018). Developing and Standardizing a Protocol for Quantitative Proton Nuclear Magnetic Resonance (1 H NMR) Spectroscopy of Saliva. J. Proteome Res. 17, 1521–1531. 10.1021/acs.jproteome.7b00847 29498859 PMC6558279

[B11] Johansson I. Witkowska E. Kaveh B. Lif Holgerson P. Tanner A. C. R. (2016). The Microbiome in Populations With a Low and High Prevalence of Caries. J. Dent. Res. 95 (1), 80–6. 10.1177/0022034515609554 PMC4700664 26442950

[B12] Koch G. (1967). Effect of Sodium Fluoride in Dentifrice and Mouthwash on Incidence of Dental Caries in Schoolchildren. Odontol. Rev. 18, 38–43.

[B13] Kressirer C. A. Smith D. J. King W. F. Dobeck J. M. Starr J. R. Tanner A. C. R. (2017). Scardovia Wiggsiae and its Potential Role as a Caries Pathogen. J. Oral. Biosci. 59, 135–141. 10.1016/j.job.2017.05.002 29104444 PMC5665406

[B14] Kursa M. B. Rudnicki W. R. (2010). Feature Selection With the Boruta Package. J. Stat. Softw. 36, 1–13. 10.18637/jss.v036.i11

[B15] Lê Cao K.-A. Martin P. G. P. Robert-Granié C. Besse P. (2009). Sparse Canonical Methods for Biological Data Integration: Application to a Cross-Platform Study. BMC Bioinf. 10:34. 10.1186/1471-2105-10-34 PMC2640358 19171069

[B16] Liaw A. Wiener M. (2002). Classification and Regression by Randomforest. R. News 2, 18–22.

[B17] Loesche W. J. Rowan J. Straffon L. H. Loos P. J. (1975). Association of Streptococcus Mutants With Human Dental Decay. Infect. Immun. 11, 1252–1260. 10.1128/IAI.11.6.1252-1260.1975 1140847 PMC415207

[B18] Mark Welch J. L. Rossetti B. J. Rieken C. W. Dewhirst F. E. Borisy G. G. (2016). Biogeography of a Human Oral Microbiome at the Micron Scale. Proc. Natl. Acad. Sci. U. S. A. 113, E791–E800. 10.1073/pnas.1522149113 26811460 PMC4760785

[B19] Marsh P. D. (1994). Microbial Ecology of Dental Plaque and its Significance in Health and Disease. Adv. Dent. Res. 8, 263–271. 10.1177/08959374940080022001 7865085

[B20] Matsui R. Cvitkovitch D. (2010). Acid Tolerance Mechanisms Utilized by Streptococcus Mutans. Future Microbiol. 5, 403–417. 10.2217/fmb.09.129 20210551 PMC2937171

[B21] Mikx F. H. M. van der Hoeven J. S. (1975). Symbiosis of Streptococcus Mutans and Veillonella Alcalescens in Mixed Continuous Cultures. Arch. Oral. Biol. 20, 407–410. 10.1016/0003-9969(75)90224-1 1096856

[B22] Mira A. (2018). Oral Microbiome Studies: Potential Diagnostic and Therapeutic Implications. Adv. Dent. Res. 29, 71–77. 10.1177/0022034517737024 29355422

[B23] Nascimento M. M. Zaura E. Mira A. Takahashi N. Ten Cate J. M. (2017). Second Era of OMICS in Caries Research: Moving Past the Phase of Disillusionment. J. Dent. Res. 96, 733–740. 10.1177/0022034517701902 28384412 PMC5480809

[B24] Neilands J. Petersson L. G. Beighton D. Svensäter G. (2012). Fluoride-Supplemented Milk Inhibits Acid Tolerance in Root Caries Biofilms. Caries Res. 46, 156–160. 10.1159/000337390 22488252

[B25] Nyvad B. Crielaard W. Mira A. Takahashi N. Beighton D. (2013). Dental Caries From a Molecular Microbiological Perspective. Caries Res. 47, 89–102. 10.1159/000345367 23207320

[B26] Oksanen J. Blanchet F. G. Kindt R. Legendre P. Minchin P. R. O’Hara R. B.. (2015). Vegan: Community Ecology Package. R Package Version 2.0-10. 2013. R Packag. Ver. 2.4–3. Available at: http://CRAN.Rproject.org/package=vegan.

[B27] Pereira J. L. Duarte D. Carneiro T. J. Ferreira S. Cunha B. Soares D.. (2019). Saliva NMR Metabolomics: Analytical Issues in Pediatric Oral Health Research. Oral. Dis. 25, 1545–1554. 10.1111/odi.13117 31077633

[B28] Quast C. Pruesse E. Yilmaz P. Gerken J. Schweer T. Yarza P.. (2013). The SILVA Ribosomal RNA Gene Database Project: Improved Data Processing and Web-Based Tools. Nucleic Acids Res. 41, D590–D596. 10.1093/nar/gks1219 23193283 PMC3531112

[B29] R Development Core Team (2016). R: A Language and Environment for Statistical Computing. R. Found. Stat. Comput. 10.1017/CBO9781107415324.004

[B30] Rohart F. Gautier B. Singh A. Lê Cao K.-A. (2017). Mixomics: An R Package for ‘Omics Feature Selection and Multiple Data Integration. PloS Comput. Biol. 13, e1005752. 10.1371/journal.pcbi.1005752 29099853 PMC5687754

[B31] Rosier B. T. Buetas E. Moya-Gonzalvez E. M. Artacho A. Mira A. (2020). Nitrate as a Potential Prebiotic for the Oral Microbiome. Sci. Rep. 10, 12895. 10.1038/s41598-020-69931-x 32732931 PMC7393384

[B32] Rosier B. T. Marsh P. D. Mira A. (2018). Resilience of the Oral Microbiota in Health: Mechanisms That Prevent Dysbiosis. J. Dent. Res. 97, 371–380. 10.1177/0022034517742139 29195050

[B33] Savorani F. Tomasi G. Engelsen S. B. (2010). Icoshift: a Versatile Tool for the Rapid Alignment of 1D NMR Spectra. J. Magn. Reson. 202, 190–202. 10.1016/j.jmr.2009.11.012 20004603

[B34] Senneby A. Davies J. Svensäter G. Neilands J. (2017). Acid Tolerance Properties of Dental Biofilms *In Vivo* . BMC Microbiol. 17, 165. 10.1186/s12866-017-1074-7 28743239 PMC5525231

[B35] Silk H. Kwok A. (2017). Addressing Adolescent Oral Health: a Review. Pediatr. Rev. 38, 61–68. 10.1542/pir.2016-0134 28148703

[B36] Simón-Soro A. Mira A. (2015). Solving the Etiology of Dental Caries. Trends Microbiol. 23, 76–82. 10.1016/j.tim.2014.10.010 25435135

[B37] Takahashi N. (2015). Oral Microbiome Metabolism. J. Dent. Res. 94, 1628–1637. 10.1177/0022034515606045 26377570

[B38] Takahashi N. Washio J. Mayanagi G. (2010). Metabolomics of Supragingival Plaque and Oral Bacteria. J. Dent. Res. 89, 1383–1388. 10.1177/0022034510377792 20924070

[B39] Teng F. Yang F. Huang S. Bo C. Xu Z. Z. Amir A.. (2015). Prediction of Early Childhood Caries *via* Spatial-Temporal Variations of Oral Microbiota. Cell Host Microbe 18, 296–306. 10.1016/j.chom.2015.08.005 26355216

[B40] Wishart D. S. Feunang Y. D. Marcu A. Guo A. C. Liang K. Vázquez-Fresno R.. (2018). HMDB 4.0: The Human Metabolome Database for 2018. Nucleic Acids Res. 46, D608–D617. 10.1093/nar/gkx1089 29140435 PMC5753273

[B41] Yamada T. Carlsson J. (1975). Regulation of Lactate Dehydrogenase and Change of Fermentation Products in Streptococci. J. Bacteriol. 124, 55–61. 10.1128/jb.124.1.55-61.1975 1176435 PMC235863

[B42] Zhang L. Sun T. Zhu P. Sun Z. Li S. Li F.. (2020). Quantitative Analysis of Salivary Oral Bacteria Associated With Severe Early Childhood Caries and Construction of Caries Assessment Model. Sci. Rep. 10, 6365. 10.1038/s41598-020-63222-1 32286402 PMC7156402

