# Supplementary material for: Bacterial Composition and Metabolomics of Dental Plaque From Adolescents
Source: Front Cell Infect Microbiol. 2021 Jul 30;11:716493. doi: 10.3389/fcimb.2021.716493 (PMC8362896; doi:10.3389/fcimb.2021.716493)
Supplement: Supplementary file 1 [file DataSheet_1.pdf]

Supplementary table 1. Baseline caries prevalence, sex and ages in Caries and Caries-Free subjects. Caries prevalence at surfaces was described as means ± SD, ranges, 95% CI and medians in permanent dentition in adolescents aged 14-18. Proximal caries lesions were diagnosed radiographically and included initial and manifest lesions and dental restorations ($D_{i+m}Fa$). An independent T-Test was used to calculate differences in ages between the two groups.

	Caries Group N=20				Caries-Free Group N=20				
	mean ± SD	range	95% CI	median	mean ± SD	range	95% CI	median	p-value
Tooth surfaces with initial caries (D_i)	8.55 ± 4.57	1-16	6.41-10.7	8.50	0	0		0	
Tooth surfaces with manifest caries (D_m)	1.60 ± 2.92	0-12	0.26-2.94	0.50	0	0		0	
Total carious tooth surfaces ($D_{i+m}F$)	17.25 ± 11.6	3-36	11.8-22.7	15.00	0	0		0	
Proximal tooth surfaces with caries ($D_{i+m+}Fa$)	11.20±7.18	3-25	7.8-14.56	8.50	0	0		0	
Ages (yr)	16.3±1.49	14-18	15.6-17.0	16.00	15.6±1.23	14-18	15-16.2		0.114
Sex (% male)	50				50				

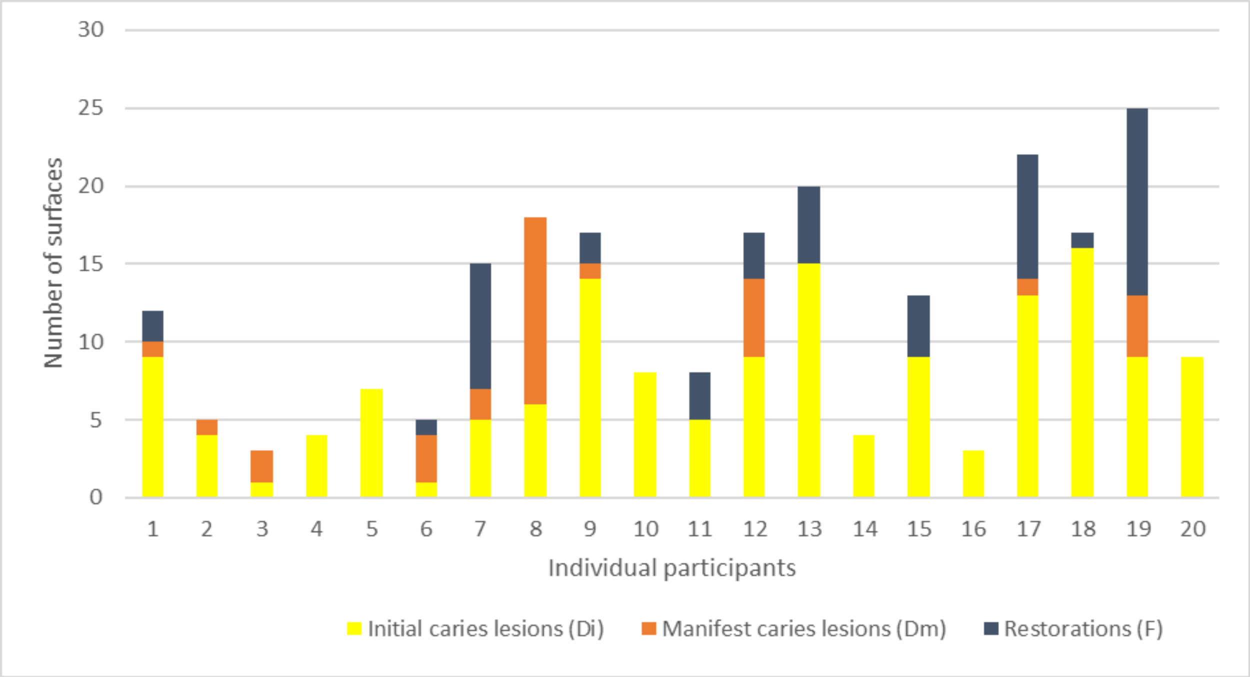

Caries prevalence at proximal surfaces. Distribution and number of initial caries lesions (Di), manifest caries lesions and restorations at proximal surfaces in twenty caries-experienced adolescents.

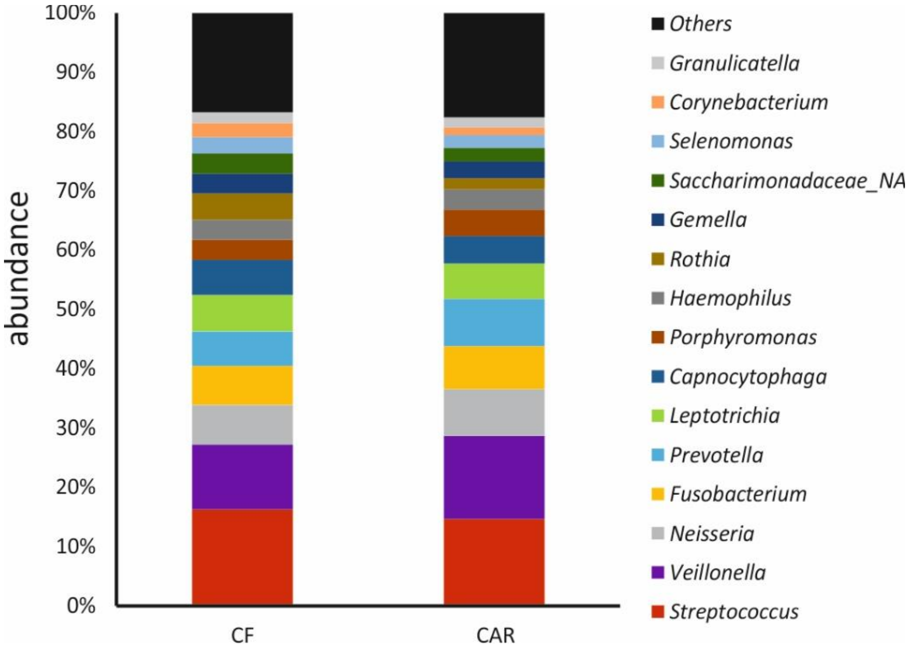

Supplementary Figure 1. Bacterial composition in dental plaque samples from caries-experienced (CAR) and caries-free (CF) Swedish adolescents. The bars show the mean abundance of the top 15 genera, while the remaining low-abundance bacteria are grouped as "Others".

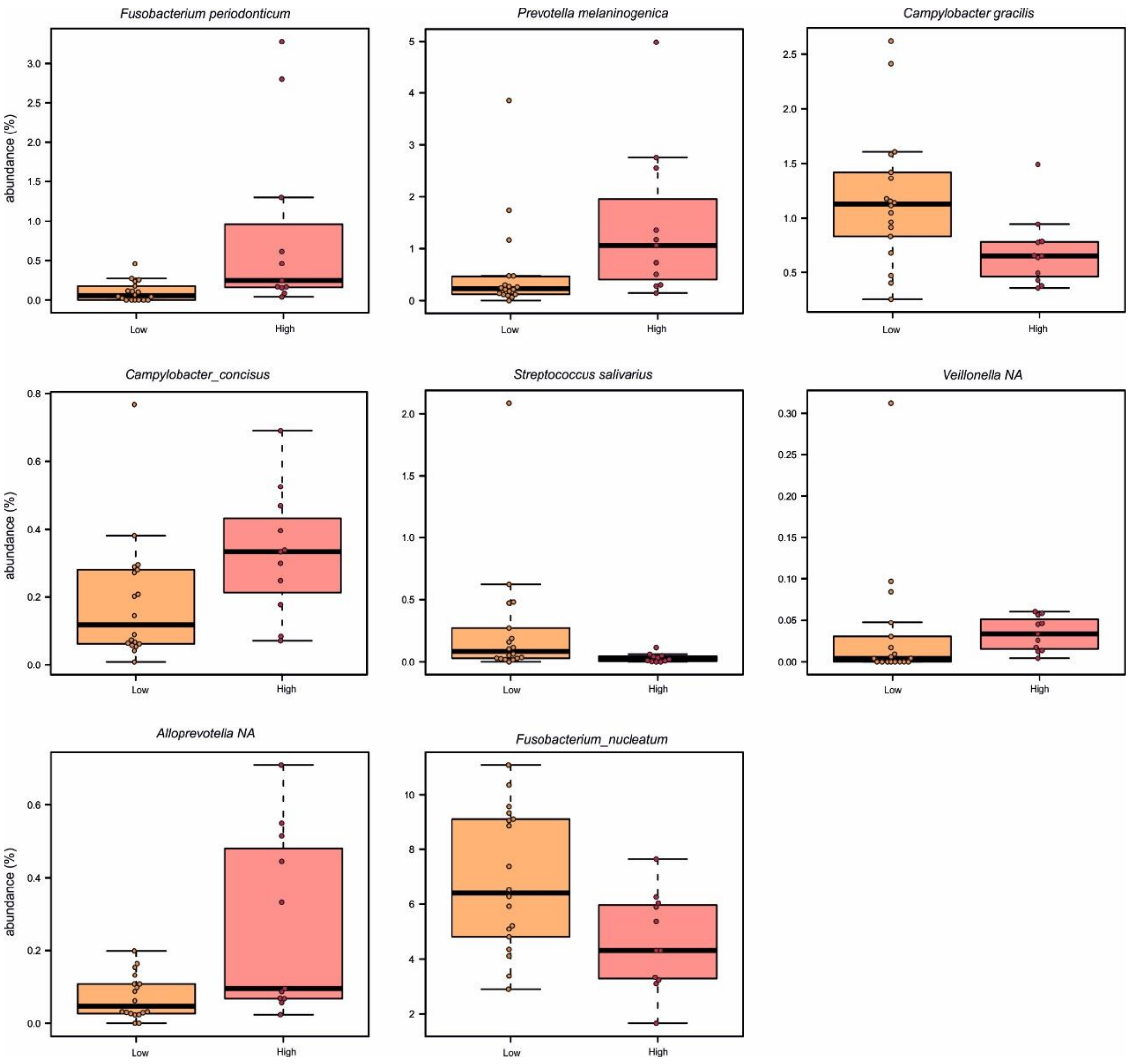

Supplementary Figure 2. Bacterial genera with differences in abundance between Low and High acid tolerance groups. Individual samples are represented as circles. All p-values (Wilcox test) are below 0.05.

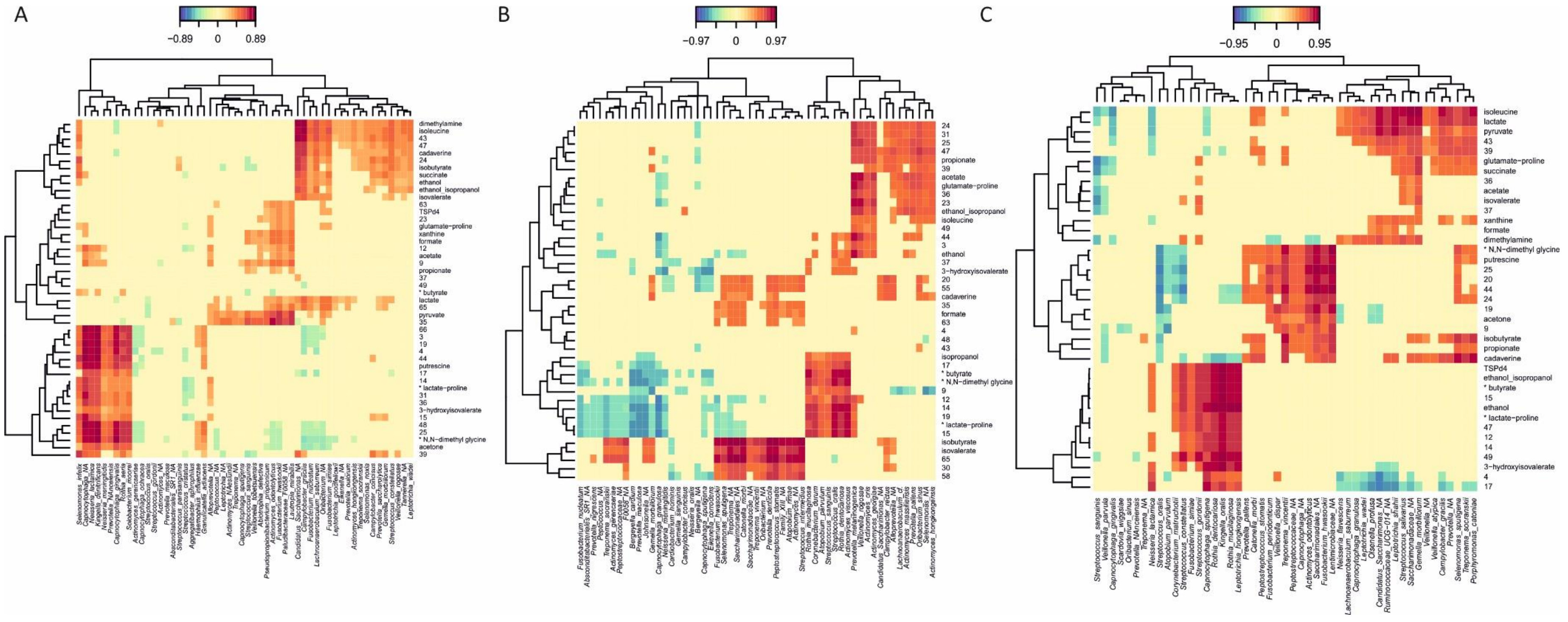

Supplementary Figure 3. Metabolomic profiles of dental plaque samples with different Acid Tolerance (AT) levels. NMR was used to quantify metabolites produced by dental plaque samples exposed to high sugar levels. The heatmaps show those metabolites with a significant correlation with bacterial species identified by Illumina sequencing of the 16S rRNA in individuals with low (A), mid (B) and high (C) acid tolerance. The degree and sign of the correlations are represented by the color code shown on top of the panels. Each bacterial species show a metabolomic profile (columns), and those profiles were clustered according to their similarity (top and left dendrograms). For those metabolites for which the NMR peaks were not unique an asterisk was added next to the putative metabolite assigned.
